# Zinc Binding to the Tyr402 and His402 Allotypes of Complement Factor H: Possible Implications for Age-Related Macular Degeneration

**DOI:** 10.1016/j.jmb.2011.03.006

**Published:** 2011-05-13

**Authors:** Ruodan Nan, Irene Farabella, Felix F. Schumacher, Ami Miller, Jayesh Gor, Andrew C.R. Martin, David T. Jones, Imre Lengyel, Stephen J. Perkins

**Affiliations:** 1 Department of Structural and Molecular Biology, Division of Biosciences, Darwin Building, University College London, Gower Street, London WC1E 6BT, UK; 2 Department of Ocular Biology and Therapeutics, UCL Institute of Ophthalmology, University College London, 11-43 Bath Street, London EC1V 9EL, UK

**Keywords:** AMD, age-related macular degeneration, FH, factor H, RPE, retinal pigment epithelium, sRPEd, subretinal pigment epithelial deposit, SCR, short complement regulator, AUC, analytical ultracentrifugation, AREDS, Age-Related Eye Disease Study, EDTA, ethylenediaminetetraacetic acid, PDB, Protein Data Bank, HSA, human serum albumin, CM, contact matrix, X-ray scattering, ultracentrifugation, molecular modelling, age-related macular degeneration, retinal pigment epithelium

## Abstract

The Tyr402His polymorphism of complement factor H (FH) with 20 short complement regulator (SCR) domains is associated with age-related macular degeneration (AMD). How FH contributes to disease pathology is not clear. Both FH and high concentrations of zinc are found in drusen deposits, the key feature of AMD. Heterozygous FH is inhibited by zinc, which causes FH to aggregate. Here, zinc binding to homozygous FH was studied. By analytical ultracentrifugation, large amounts of oligomers were observed with both the native Tyr402 and the AMD-risk His402 homozygous allotypes of FH and both the recombinant SCR-6/8 allotypes with Tyr/His402. X-ray scattering also showed that both FH and SCR-6/8 allotypes strongly aggregated at > 10 μM zinc. The SCR-1/5 and SCR-16/20 fragments were less likely to bind zinc. These observations were supported by bioinformatics predictions. Starting from known zinc binding sites in crystal structures, we predicted 202 putative partial surface zinc binding sites in FH, most of which were in SCR-6. Metal site prediction web servers also suggested that SCR-6 and other domains bind zinc. Predicted SCR-6/8 dimer structures showed that zinc binding sites could be formed at the protein–protein interface that would lead to daisy-chained oligomers. It was concluded that zinc binds weakly to FH at multiple surface locations, most probably within the functionally important SCR-6/8 domains, and this explains why zinc inhibits FH activity. Given the high pathophysiological levels of bioavailable zinc present in subretinal deposits, we discuss how zinc binding to FH may contribute to deposit formation and inflammation associated with AMD.

## Graphical Abstract

**Figure:**
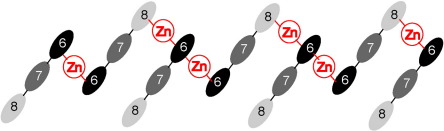

## Introduction

The complement system of the innate immune system is activated by the alternative pathway through the low-level spontaneous hydrolysis of C3 to form C3u (also known as C3_H2O_), which leads to a positive-feedback amplification of C3 cleavage to form activated C3b.^1–3^ While complement is targeted against pathogenic bacteria, complement-mediated host cell damage is prevented by the complement regulator factor H (FH), which acts as a cofactor for factor I to cleave C3b, competes with factor B to inhibit the formation of the C3 convertase C3bBb, and accelerates the decay of C3bBb.^4^ FH is constructed from 20 short complement regulator (SCR) domains, each about 61 residues in length.^5^ The SCR domains in heterozygous and homozygous FHs form a partially folded-back structure in solution.^6–8^ FH binds to glycosaminoglycans on the host cell surface through its C-terminal region and to SCR-7, and this is followed by the decay-accelerating and cofactor activity of FH against C3b through the N-terminal region.^9–11^ FH binds to C3b at SCR-1/4 and SCR-19/20.^12^ FH also binds to other ligands such as C-reactive protein, M-protein of *Streptococci* bacterium, and FH-binding protein of *Neisseria meningitides*.^13–16^ FH also self-associates to form dimer, trimer, and higher oligomers.^8,17–19^ FH oligomerisation is strongly promoted by zinc, followed by copper.^20,21^ X-ray scattering and analytical ultracentrifugation (AUC) experiments showed that pooled native heterozygous FH aggregates strongly in the presence of ≥ 10 μM zinc or copper, and this is matched by the decrease in fluid-phase FH activity.^21^

FH is genetically associated with age-related macular degeneration (AMD), the most common cause of blindness in the elderly in the Western population.^22–26^ FH is also involved with atypical haemolytic uraemic syndrome, membranoproliferative glomerulonephritis type II, and Alzheimer's disease.^27–29^ Even with the analysis of over 100 genetic alterations, the molecular role of FH in these diseases is still unclear.^28^ This lack of knowledge is attributed in part to the multivalent weak affinities of FH for its ligands, which makes experimental studies difficult.^30,31^ A hallmark of early AMD is the appearance and growth of subretinal pigment epithelial deposits (sRPEds) that develop within Bruch's membrane, an extracellular matrix layer interposed between the retinal pigment epithelium (RPE) and the choroidal vasculature.^32–34^ sRPEds contain oxidised lipids, carbohydrates, cellular materials, and over 140 aggregated proteins including FH and other complement components. FH is secreted by RPE cells or is delivered by the choroidal blood circulation.^22,26,35,36^ Complement-activation-related inflammation has been linked with the formation of sRPEds.^22,26,37^ A Tyr402His polymorphism in FH SCR-7 is associated with 50% of AMD cases.^23–26^ The SCR-6/8 His402 allotype shows slightly greater self-association than the Tyr402 allotype.^8,17^ While the binding of C-reactive protein to FH was recently disputed, the most recent study confirmed that the two proteins interact with each other and that the SCR-6/8 His402 allotype binds more weakly to C-reactive protein than the Tyr402 allotype.^4,13,30^ The two FH allotypes showed similar cofactor activity in C3b degradation.^12,38^ The effect of glycosaminoglycans on these two FH allotypes remains unclear, sometimes with conflicting results.^39^

Zinc is abundant in the human retina.^40^ It is vital for retinal cell survival and the functioning of antioxidant enzymes and the visual cycle, while an excess of zinc can be detrimental and exacerbate neuronal damage.^41^ Under oxidative stress during light exposure, the secretion of zinc from the zinc-rich RPE cells is elevated.^41^ Millimolar concentrations of zinc have been found in sRPEds and Bruch's membrane in AMD, suggesting that the pathological release of zinc from surrounding tissues like the RPE–choroid complex might be involved in deposit formation.^40,42^ Studies of the effect of zinc supplements on reducing the development of AMD gave different results. Based on the premise that zinc is an antioxidant in retina, the large Age-Related Eye Disease Study (AREDS) indicated that supplements with zinc alone or with zinc together with vitamins C and E and β carotene reduce the risk for progression to advanced AMD.^43,44^ Interestingly, a follow-up study found that the treatment response to the AREDS-recommended zinc supplement was influenced by the Tyr402His FH polymorphism. A smaller reduction in the progression to AMD to advanced stage disease was found in patients with the FH His402 allotype than in those with the FH Tyr402 allotype.^45^

Zinc binding sites show a tetrahedral coordination geometry at which the binding site is often formed from surface His, Asp, Glu, and Cys residues.^46^ When these zinc sites are found between two protein surfaces, between one and three residues at each surface contribute to a tetrahedral coordination involving four residues. The Tyr402His polymorphism potentially provides a new His zinc-binding ligand within the SCR-6/8 region that binds to heparin and C-reactive protein. To clarify whether the two allotypes exhibit different zinc binding properties, we studied the homozygous FH Tyr402 and His402 allotypes and the two allotypes of recombinant SCR-6/8 titrated with zinc. By AUC and synchrotron X-ray scattering, we showed that zinc interacts with both homozygous allotypes of FH. We also studied the effect of zinc on the other functionally important SCR-1/5 (binds C3b) and SCR-16/20 (binds C3b, C3d, C-reactive protein, and heparin) regions. Bioinformatics predictions supported the observation of zinc binding to SCR-6/8 and less so to SCR-1/5 or SCR-16/20. We discuss the molecular implications of our results with FH and zinc for complement regulation and sRPEd formation in AMD.

## Results and Discussion

### Sedimentation velocity of the complexes of the two FH and SCR-6/8 allotypes with zinc

The homozygous full-length FH and recombinant SCR-6/8 allotypes (Tyr402 and His402) were purified (Materials and Methods). During this study, the two FH allotypes were studied at a concentration of 0.7–0.8 mg/ml (4.6–5.2 μM) in order to be comparable in concentration with the physiological FH range of 0.235–0.810 mg/ml in serum.^28^ The SCR-6/8 allotypes were studied at 0.3 mg/ml (14.6 μM), which was the lowest concentration that produced analysable data. Both proteins were studied in Hepes buffer (Materials and Methods) in order to avoid the precipitation of zinc with phosphate. Both were titrated with 0–600 μM ZnSO_4_ in ultracentrifugation and scattering experiments.

**Fig. 1:**
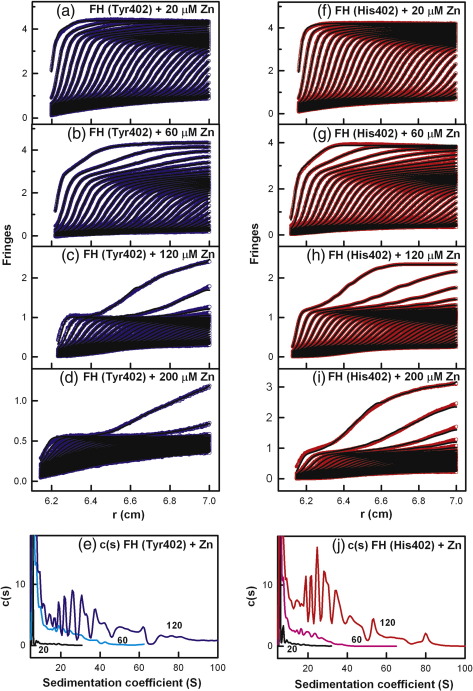
Sedimentation velocity analyses of the two FH allotypes titrated with zinc. Both allotypes were studied at 0.7 mg/ml. In the sedimentation boundary fits, only every 10th scan is shown for clarity. (a–d) The fits corresponding to the FH Tyr402 allotype (026II; blue) was titrated with zinc concentrations of 20 μM, 60 μM, 120 μM, and 200 μM in that order. (f–i) The fits corresponding to the FH His402 allotype (027BA; red) was titrated with the same zinc concentrations. (e and j) The averaged *c*(*s*) sedimentation coefficient distribution analyses from each of (a) to (c) and (f) to (h) are shown for the FH Tyr402 and FH His402 allotypes, respectively. The height of the FH monomer peak close to 5.7 S is normalised to 100 for clarity. The zinc concentrations (μM) are denoted numerically.

**Fig. 2:**
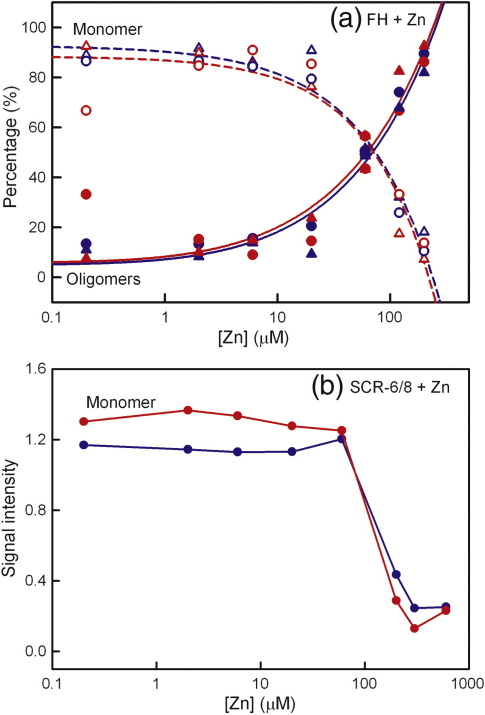
Dependence of the monomer and oligomers of FH and SCR-6/8 on zinc concentrations. (a) The values from *c*(*s*) integrations correspond to the averages obtained at 50,000 rpm and 60,000 rpm. The oligomer data were fitted to a two-parameter power function, *y* = *a* × (1 + *x*)^*b*^, and the monomer data were fitted to the function *f* = *y*0 + *a* × *x^b^*. Oligomers are denoted by filled symbols, and monomers are denoted by open symbols for four experiments with two FH Tyr402 (026II, 022LM, blue, ●, ○, ▲, Δ) and two FH His402 (027BA, 032KO, red, ●, ○, ▲, Δ) allotypes. (b) The proportions of monomer of the Tyr402 (blue) and His402 (red) allotypes of SCR-6/8 are shown.

AUC studies the sedimentation behaviour of macromolecules on subjecting these to a high centrifugal force in order to determine their sizes and shapes.^47^ Sedimentation velocity experiments were performed using speeds of up to 60,000 rpm on two freshly prepared Tyr402 and two His402 FH allotypes at concentrations of 0.7–0.8 mg/ml, each being titrated with zinc from 0.2 μM to 200 μM. Similar sets of rapidly moving sedimentation boundaries were observed for both allotypes when [Zn] increased. Good fits to the sedimentation boundaries were obtained in all cases using sedimentation coefficient distribution analyses *c*(*s*) (Fig. 1a–d and f–i). At all [Zn] values, the FH monomer was observed at a mean value of 5.67 ± 0.05 S for the Tyr402 allotype and at 5.74 ± 0.07 S for the His402 allotype at 50,000 rpm. This indicated the presence of zinc-free FH even with a 40-fold excess of zinc, meaning that the binding of zinc is weak. For both allotypes with [Zn] between 0.2 and 20 μM, additional peaks that corresponded to similar small amounts of oligomers from dimers to nonamers were visible from 7 S to 30 S that resembled those for zinc-free FH.^7,8,19^ For both allotypes with [Zn] between 60 and 200 μM, sizeable amounts of rapidly sedimenting species were observed with increased *s*_20,w_ values of up to 100 S in the *c*(*s*) distributions (Fig. 1e and j). The proportions of oligomers of the FH Tyr402 and His402 allotypes were derived by integration of the *c*(*s*) size distribution analyses. No differences in the proportions of the FH monomer and oligomers at each zinc concentration were seen between the allotypes. Like heterozygous pooled native FH,^21^ both the Tyr402 and the His402 allotypes form heavy oligomers with zinc and share similar dependences on [Zn] (Fig. 2a).

The sedimentation boundaries showed that differential ranges of *S* values for the zinc-induced FH oligomers were formed with the two allotypes. For the Tyr402 and His402 allotypes with zinc in high excess at 200 μM, the oligomer peaks ranged in size with variable amounts in repeated runs with *S* values in excess of 100 S, indicating their sensitivity to the presence of zinc when zinc was in excess. These differences were reflected in Fig. 1a–d and f–i, where the upper part of the boundaries sedimented more rapidly for the Tyr402 and His402 allotypes at all zinc concentrations. In addition, with the use of the homozygous FH proteins whose genetic heterogeneity is much reduced compared to the pooled FH used in earlier studies, it was noteworthy that the *c*(*s*) peaks show no sequence of well-resolved peaks with well-defined *S* values similar to the individual peaks 2–9 seen in zinc-free full-length FH.^7,8,19^ These further observations suggested that a broad range of zinc-induced FH oligomers of different sizes was formed through the cross-linking of different weak zinc binding sites at the surface of FH.

**Fig. 3:**
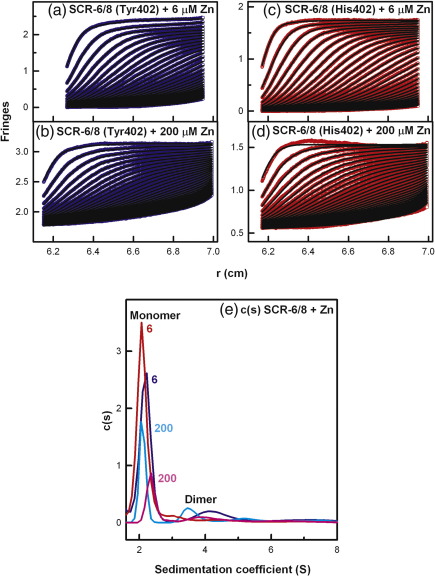
Sedimentation velocity analyses of the two SCR-6/8 allotypes titrated with zinc. Both allotypes were studied at 0.3 mg/ml. In the sedimentation boundary fits, only every 10th scan is shown for clarity. (a and b) The fits for the SCR-6/8 Tyr402 allotype (blue) titrated with zinc at 6 μM and 200 μM. (c and d) The fits for the SCR-6/8 His402 allotype (red) titrated with zinc at 6 μM and 200 μM. (e) The *c*(*s*) sedimentation coefficient distribution analyses for the Tyr402 allotype with 6 μM (blue) and 200 μM (cyan) zinc, and the His402 allotype with 6 μM (red) and 200 μM (pink) zinc. The *c*(*s*) distributions are labelled with their zinc concentration values (μM). The peak at about 2.1 S corresponds to the monomer, and that at about 4.0 S is the dimer.

In order to clarify the molecular basis for zinc-induced FH oligomers, we performed sedimentation velocity experiments at 50,000 rpm on the Tyr402 and His402 allotypes of the SCR-6/8 fragment at 0.3 mg/ml (14.6 μM) titrated with zinc from 0.2 μM to 600 μM. Similar sedimentation boundaries were observed for the two allotypes of SCR-6/8 at each [Zn] (Fig. 3a–d). In contrast to full-length FH, no rapidly sedimenting larger species were detected in the size distribution *c*(*s*) fits at any [Zn]. The *c*(*s*) analyses consistently showed a major monomer peak at 2.12 ± 0.09 S and a minor dimer peak at 4.03 ± 0.20 S for the Tyr402 allotype and, likewise, two peaks at 2.19 ± 0.08 S and 3.95 ± 0.16 S for the His402 allotype (Fig. 3e). This agrees with the previous observation of SCR-6/8 monomer and dimer by sedimentation velocity, where the assignment of monomer and dimer was made based on the molecular weights derived from the *c*(*s*) analyses.^17^ However, with [Zn] above 60 μM, the signal intensity of the monomer peak for both allotypes decreased significantly with an increase in [Zn] (Figs. 2b and 3e). This indicates that, at [Zn] above 60 μM, zinc induced the formation of aggregated SCR-6/8, which were sufficiently large to sediment to the bottom of the sample cell before the first boundary scan was recorded. While the His402 allotype showed slightly greater aggregation than the Tyr402 allotype, this may result from experimental variability in quantifying the different sedimenting species. Overall, it was noteworthy that SCR-6/8 aggregated at the same [Zn] value that caused large FH oligomers to form. This indicated that the major binding sites for zinc in full-length FH were located within SCR-6/8.

**Fig. 4:**
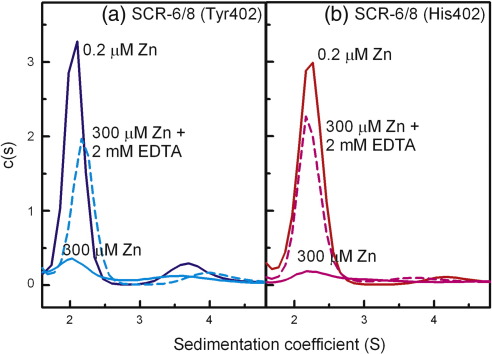
The effect of EDTA on the interactions between the SCR-6/8 allotypes and zinc using sedimentation velocity. (a) The *c*(*s*) distribution analyses are shown for the Tyr402 allotype in the presence of 0.2 μM zinc (blue continuous line), 300 μM zinc (cyan continuous line), and 300 μM zinc and 2 mM EDTA (cyan broken line). (b) The *c*(*s*) distribution analyses are shown for the His402 allotype in the presence of 0.2 μM zinc (red continuous line), 300 μM zinc (pink continuous line), and 300 μM zinc and 2 mM EDTA (pink broken line).

The reversibility of the zinc-induced aggregation of the SCR-6/8 allotypes was studied by adding 2 mM ethylenediaminetetraacetic acid (EDTA) to the ultracentrifuge cell containing 0.3 mg/ml SCR-6/8 and 300 μM zinc. After mixing, we immediately re-ran the sedimentation velocity experiment at 50,000 rpm on this cell again. Even though the size distribution *c*(*s*) analyses showed that the monomer peak for both allotypes of SCR-6/8 with 300 μM zinc was much reduced, the addition of EDTA caused a significant increase in the monomer peak intensity for both allotypes (Fig. 4). This showed that the zinc-induced aggregation of SCR-6/8 was reversible, in good agreement with the reversibility of the zinc-induced oligomers of heterozygous pooled FH by adding EDTA.^21^

**Fig. 5:**
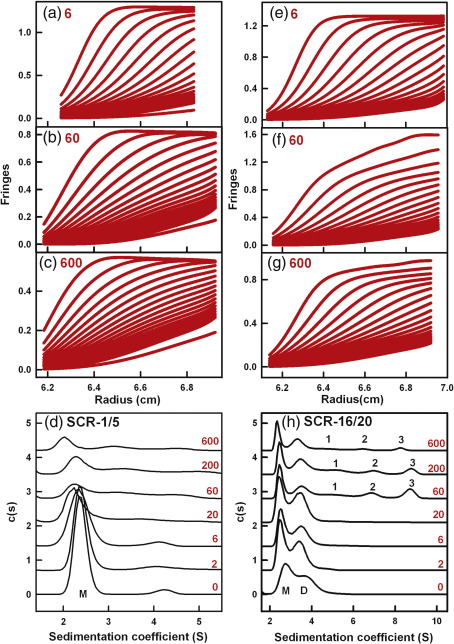
Sedimentation velocity analysis of SCR-1/5 and SCR-16/20 titrated with zinc. Red numbers indicate zinc concentrations (μM). (a–c) For SCR-1/5, the boundary fits at three zinc concentrations are shown for only every 24th scan out of 480 scans for reason of clarity. (d) Size distribution *c*(*s*) analyses for SCR-1/5 at seven zinc concentrations are shown. M indicates the monomer position. The graphs were displaced vertically in units of 0.5 for reason of clarity. (e–g) For SCR-16/20, the boundary fits at three zinc concentrations are shown for only every 22nd scan out of 440 scans. (h) Size distribution *c*(*s*) analyses for SCR-16/20 at seven zinc concentrations are shown. M and D indicate the monomer and dimer peaks, respectively. Weak emerging signals are denoted by 1, 2, and 3. Graphs are displaced vertically in units of 0.5 for reason of clarity.

Additional ultracentrifugation experiments with the SCR-1/5 and SCR-16/20 fragments in the presence of zinc were performed as controls of the SCR-6/8 experiments. SCR-1/5 is less soluble at 0.3 mg/ml when expressed, compared to SCR-6/8 and SCR-16/20. As [Zn] increased, SCR-1/5 aggregated in the same manner as SCR-6/8 at a similar [Zn] value to that for SCR-6/8, and this may reflect its lower solubility. This was visible from both the reduced intensities of the fringes as [Zn] increased and the marked reduction in the monomer peak intensity in the *c*(*s*) plot (Fig. 5a–d). In contrast to this, SCR-16/20 is more soluble than SCR-1/5 and SCR-6/8, and SCR-16/20 remained in solution as [Zn] increased with a smaller reduction in the number of observed fringes at 600 μM. Here, the *c*(*s*) plot showed a smaller reduction in the sizes of the monomer and dimer peaks, while at the same time, as many as three other species were observed (peaks 1, 2, and 3 in Fig. 5e–h). These experiments showed that other zinc-induced oligomers formed in two other regions of FH. However, unlike SCR-6/8, both SCR-1/5 and SCR-16/20 possessed His_6_-tags that may comprise potential zinc binding sites.^18^ This possibility was evaluated by further zinc titrations in which a 2:1 molar ratio of nickel (17 μM) was present to saturate its binding site at the His_6_-tag, to which nickel binds more tightly with a dissociation constant of 1 μM than zinc.^48^ The resulting *c*(*s*) plots showed that no SCR-1/5 or SCR-16/20 aggregates were seen and that only the monomer or monomer/dimer peaks that were similar to those observed for zinc-free protein were observed. These final experiments indicate either that the His_6_-tag represented an oligomeric zinc binding site and this is eliminated by the stronger binding of nickel to this or that nickel competes with zinc for weak interaction sites in FH. The first explanation is preferred because nickel has no strong effect on FH oligomer formation.^21^ Three control experiments with full-length FH His402 in the presence of 200 μM zinc and 20 μM nickel, 200 μM zinc, or 20 μM nickel were performed to complete these studies. This showed that, in the presence of nickel, zinc still causes full-length FH to aggregate in similar quantities, even though zinc-induced oligomer formation in SCR-1/5 and SCR-16/20 was individually blocked by nickel. Even though these experiments are cautionary in indicating that other weak zinc binding sites in FH may exist, it was concluded that a major region for zinc-binding to FH lies in SCR-6/8.

### X-ray scattering of the two allotypes of FH and SCR-6/8 with zinc

X-ray scattering is a diffraction method used to study the solution structures of macromolecules in random orientations.^49^ The effect of zinc on the structures of freshly purified two Tyr402 and two His402 allotypes of full-length FH and the two allotypes of FH fragment SCR-6/8 was investigated by synchrotron X-ray scattering. FH was studied at 0.9 mg/ml (5.8 μM) in order to be comparable with physiological FH concentrations, while SCR-6/8 was studied at 0.2 mg/ml (9.7 μM), this being the lowest concentration of SCR-6/8 that produced analysable data. Each FH or SCR-6/8 sample was titrated with 0 μM to 600 μM ZnSO_4_. The scattering data *I*(*Q*) showed excellent signal-to-noise ratios and no detectable effect from radiation damage.

**Fig. 6:**
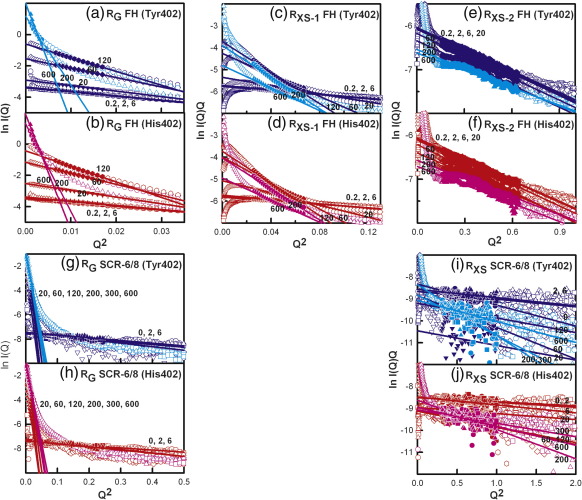
X-ray Guinier analyses of the full-length FH and SCR-6/8 allotypes titrated with zinc. In all panels, the open symbols correspond to the experimental data and the filled symbols correspond to those used for the *R*_G_ and *R*_XS_ straight line fits. Data correspond to one FH Tyr402 allotype (018DK) in (a), (c), and (e) and one FH His402 allotype (030TJ) in (b), (d), and (f), both at 0.9 mg/ml, and SCR-6/8 Tyr402 (g and i) and SCR-6/8 His402 (h and j), both at 0.2 mg/ml. Data for the Tyr402 allotype of FH and SCR-6/8 are shown in blue from 0.2 μM to 120 μM ZnSO_4_ and in cyan from 200 μM to 600 μM ZnSO_4_. Data for the His402 allotype of FH and SCR-6/8 are shown in red from 0.2 μM to 120 μM ZnSO_4_ and in pink from 200 μM to 600 μM ZnSO_4_. (a and b) Guinier *R*_G_ plots of ln *I*(*Q*) *versus Q*^2^ for FH Tyr402 and FH His402 allotypes titrated with ZnSO_4_ concentrations of 0.2 μM (O), 2 μM (□), 6 μM (Δ), 20 μM (▿), 60 μM (◊), 120 μM (⎔), 200 μM (O), and 600 μM (Δ). The *Q* fit ranges were 0.08–0.13 nm^− 1^ for 0.2 μM to 120 μM ZnSO_4_ and 0.03–0.16 nm^− 1^ for 200 μM to 600 μM ZnSO_4_. The zinc concentrations are numerically labelled as shown. (c and d) The corresponding Guinier cross-sectional *R*_XS-1_ fits of ln *I*(*Q*)*Q versus Q*^2^ for the two FH allotypes with zinc using a *Q* range of 0.16–0.26 nm^− 1^. (e and f) The corresponding Guinier cross-sectional *R*_XS-2_ fits for the two FH allotypes with zinc using a *Q* range of 0.4–0.8 nm^− 1^. (g and h) Guinier *R*_G_ plots for the SCR-6/8 Tyr402 and His402 allotypes at 0.2 mg/ml titrated with ZnSO_4_ at concentrations of 0 μM (○), 2 μM (□), 6 μM (Δ), 20 μM (▿), 60 μM (◊), 120 μM (⎔), 200 μM (O), 300 μM (□), and 600 μM (Δ). The *Q* fit ranges were 0.16–0.5 nm^− 1^ for 0.2 μM to 120 μM ZnSO_4_ and 0.06–0.11 nm^− 1^ for 200 μM to 600 μM ZnSO_4_. (i and j) The corresponding Guinier cross-sectional *R*_XS_ fits for the SCR-6/8 allotypes using a *Q* range of 0.55–1.0 nm^− 1^.

**Fig. 7:**
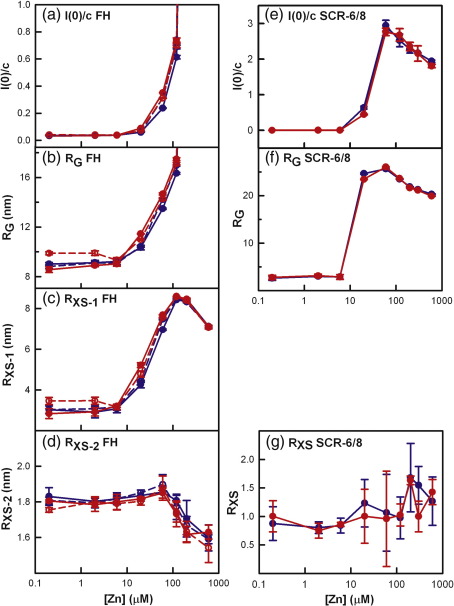
Dependence of the *R*_G_, *I*(0)/*c*, and *R*_XS_ values of FH and SCR-6/8 on the zinc concentration. In all cases, the Tyr402 allotype is shown in blue, and the His402 allotype is shown in red. Each value was measured in quadruplicate and averaged, and statistical error bars are shown where visible. In (a) to (d), the four titrations correspond to two pairs of FH Tyr402 allotypes (018DK and 016CC) and FH His402 allotypes (030TJ and 033NV) at 0.9 mg/ml titrated with zinc from 0.2 to 600 μM (each pair shown as broken and continuous lines). In (e) to (g), the titrations correspond to the two SCR-6/8 allotypes titrated with the same zinc concentration range from 0.2 to 600 μM.

Guinier fits at low *Q* values (where *Q *= 4π sin θ/λ; 2θ,  scattering angle; λ,  wavelength) give the radius of gyration (*R*_G_), which monitors the degree of elongation of the protein, and the *I*(0)/*c* value, which is proportional to the relative molecular mass (Fig. 6a and b).^49,50^ At larger *Q* values, Guinier fits give the cross-sectional radius of gyration *R*_XS_, which monitors the structural proximity relationships between non-neighbouring SCR domains (*R*_XS-1_) and neighbouring SCR domains (*R*_XS-2_) (Fig. 6c–f).^6^ The overall folded-back SCR domain structure of FH leads to the *R*_XS-1_ value, while the localized, extended structural arrangement of SCR domains leads to the *R*_XS-2_ value. In our previous study of heterozygous and homozygous FHs, the presence of oligomers caused concentration-dependent increases in the values of *R*_G_ and *R*_XS-1_.^8,19^ Here, at the lowest zinc concentration of 0.2 μM, the mean *R*_G_ and *R*_XS-1_ values were 8.91 ± 0.17 nm and 3.03 ± 0.17 nm, respectively, for the Tyr402 FH allotype and 9.23 ± 0.72 nm and 3.15 ± 0.39 nm, respectively, for the His402 FH allotype (Fig. 7b and c). Both the *R*_G_ and the *R*_XS-1_ values were similar to those for heterozygous FH at 0.9 mg/ml without zinc^19^ and were the same within error of the corresponding values for zinc-free homozygous FH at 1.1 mg/ml.^8^ When both the Tyr402 and the His402 FH allotypes were titrated with zinc of up to 6 μM, the scattering curves *I*(*Q*) remained unchanged (Fig. 6a–d), and the Guinier values remained unchanged (Fig. 7a–c). For [Zn] from 20 μM to 600 μM, the *I*(*Q*) intensities increased significantly at low *Q* and decreased at high *Q* as the result of oligomer formation (Fig. 6a–f). The Guinier *I*(0)/*c* and *R*_G_ values increased significantly (Fig. 7a and b), although the *R*_XS-1_ and *R*_XS-2_ values decreased at [Zn] above 200 μM as the result of the decrease in the intensity of *I*(*Q*) in the *Q* ranges used for the *R*_XS_ determinations (Figs. 6c–f and 7c and d). The changes for both homozygous FH allotypes are comparable with previous observations for heterozygous native FH,^19^ indicating again that FH heterogeneity is not responsible for these observations. For [Zn] between 20 μM and 120 μM, the two His402 allotypes showed slightly higher Guinier *I*(0)/*c*, *R*_G_, and *R*_XS-1_ values than the two Tyr402 allotype (Fig. 7a–c). If this is significant, this would indicate that the His402 allotype formed more oligomers than the Tyr402 allotype; however, the difference is not considered to be large enough.

To test whether the Tyr402 and His402 allotypes of SCR-6/8 formed zinc-induced oligomers, we titrated both of them with [Zn] from 0.2 to 600 μM. For SCR-6/8 at 0.2 mg/ml with 0.2 μM zinc, the Guinier *R*_G_ and *R*_XS_ values were 2.63 ± 0.46 nm and 0.88 ± 0.29 nm, respectively, for the Tyr402 allotype, which were similar to values of 2.79 ± 0.47 nm and 1.00 ± 0.27 nm for the His402 allotype. Those Guinier values are slightly less but within error of the experimental *R*_G_ and *R*_XS_ values of 3.26–3.35 nm and 1.04 nm, respectively, for the zinc-free Tyr402 allotype and 3.12–3.21 nm and 1.15 nm (± 0.06–0.20 nm) for the zinc-free His402 allotype.^17^ For [Zn] between 0.2 μM and 6 μM, the scattering curves *I*(*Q*) were unchanged for both allotypes (Figs. 6g–j and 7e–g). At 20 μM [Zn], the *I*(*Q*) curves of both allotypes underwent dramatic changes in which the intensities sharply increased at low *Q* and decreased rapidly at large *Q* (Fig. 6g–j), and these changes resulted in much increased Guinier *I*(0)/*c* and *R*_G_ values (Fig. 7e and f). Above 20 μM [Zn], the *I*(*Q*) intensities continued to rise at very low *Q* (Fig. 6g and h). The Guinier *I*(0)/*c* and *R*_G_ values peaked at [Zn] of 60 μM and declined at higher [Zn] values (Fig. 7e and f). These changes showed that both allotypes of SCR-6/8 interacted with zinc starting from 10 μM, which is the same starting concentration as that of the FH interaction with zinc. Unlike the FH–zinc oligomers, which remained in solution, the changes observed for SCR-6/8 caused this to aggregate, and this accounted for the apparent decline in the *I*(0)/*c* and *R*_G_ above 60 μM. Like full-length homozygous FH, no significant difference was observed for the zinc-induced oligomerisation of the Tyr402 and His402 allotypes of SCR-6/8.

**Fig. 8:**
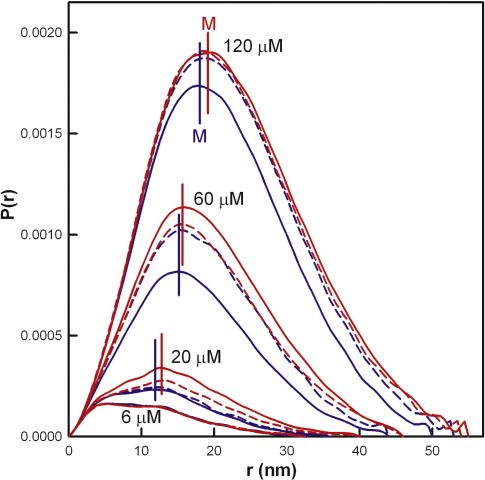
Dependence of the distance distribution function *P*(*r*) of the FH allotypes on zinc concentration. The *P*(*r*) curves were calculated from the scattering curves of the two FH Tyr402 allotypes (018DK and 016CC; blue) and two FH His402 allotypes (030TJ and 033NV; red). From bottom to top, the zinc concentrations were 6 μM, 20 μM, 60 μM, and 120 μM (labelled). The average value of the most frequently occurring distance *M* for each allotype at each zinc concentration is marked by vertical straight lines.

The distance distribution function *P*(*r*) of the two FH allotypes provided shape information in real space on the zinc-induced FH oligomers. The *P*(*r*) curve summarises the distances between all pairs of atoms within FH and gives the most frequently occurring distance *M* from the position of the peak maximum, the maximum length *L* from the point at which *P*(*r*) becomes 0 at large *Q*, and an independent calculation of the *R*_G_ and *I*(0) values for comparison with the Guinier determinations (not shown).^49^ At [Zn] of up to 6 μM, the shape of the *P*(*r*) curves remained unchanged with an increase in [Zn] and was indistinguishable between the Tyr402 and the His402 FH allotypes (Fig. 8). The length *L* of both allotypes was 32 nm, and the maximum *M* values were at 5.36 ± 0.11 nm and 5.20 ± 0.11 nm for the Tyr402 and His402 allotypes. These values are in good agreement with previous observations for heterozygous FH with zinc and showed that FH has a folded-back domain structure with an overall length *L* much reduced from a maximum length of 73 nm if its domains had been fully extended in their arrangement.^21^ Also similar to previous observations, the intensity of the *P*(*r*) curves and the *L* and *M* values for both allotypes increased with an increase in [Zn] above 6 μM, which indicates that zinc-induced oligomerisation occurred with both the Tyr402 and the His402 allotypes and was not dependent on heterogeneity (Fig. 8). The increase in *L* values showed that the oligomers had increased in size compared to the monomer. For [Zn] at 20 μM, 60 μM, and 120 μM, the shapes of the *P*(*r*) curves were similar between the two allotypes, indicating that oligomers that are similar in size and conformation had formed under these conditions (Fig. 8). The *P*(*r*) intensities and *M* values were slightly higher for the His402 allotype than for the Tyr402 allotype, which is consistent with the Guinier analyses; however, the difference is not considered to be significant (Fig. 8). When the *P*(*r*) analyses were repeated for the SCR-6/8 allotypes with [Zn] of up to 6 μM, *L* was determined to be 9 nm for both allotypes, and the curves were indistinguishable between the two allotypes (data not shown). The *P*(*r*) curves were similar in appearance to those for SCR-6/8 without zinc.^17^ Above 6 μM zinc, the aggregation of SCR-6/8 made further *P*(*r*) analyses impossible. The lack of difference between the SCR-6/8 allotypes indicated that His402 had no effect on zinc-induced oligomerisation compared to Tyr402.

### Fluid-phase activity assays of two allotypes of full-length FH complexes with zinc

**Fig. 9:**
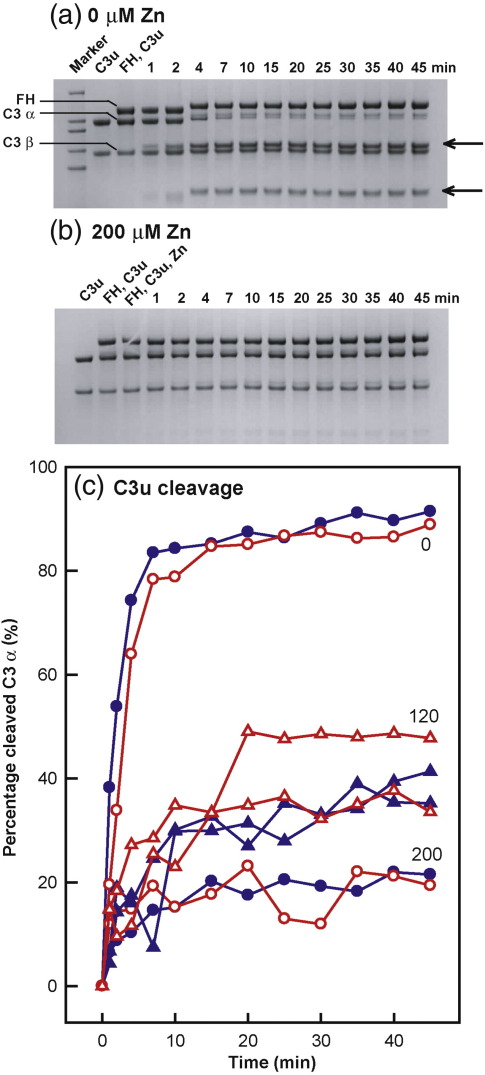
Cleavage of fluid-phase C3u by FI and the two FH allotypes in the presence of zinc. (a) Reducing SDS-PAGE analysis of C3u cleavage by factor I and the FH Tyr402 allotype (013HH) in the absence of zinc. Lane 1, High Mark™ Prestained High Molecular Weight Standard; lane 2, 0.3 mg/ml C3u; lane 3, 0.3 mg/ml C3u and 0.3 mg/ml FH; lanes 4–15 correspond to lane 3 with 0.003 mg/ml factor I added with the reaction times in minutes as labelled. FH and the α-chain and β-chain of C3u are arrowed on the left, and the α-chain cleavage products are arrowed on the right. (b) Reducing SDS-PAGE analysis of C3u cleavage by factor I and the FH Tyr402 allotype (013HH) with 200 μM zinc. Lane 1, 0.3 mg/ml C3u; lane 2, 0.3 mg/ml C3u and 0.3 mg/ml FH; lane 3, 0.3 mg/ml C3u, 0.3 mg/ml FH, and 200 μM zinc; lanes 4–15 correspond to lane 3 with the reaction times in minutes as labelled. (c) The percentage cleavage of the C3 α-chain by factor I and the FH Tyr402 and His402 allotypes is shown as a function of reaction time. The [Zn] values are labelled as shown. The Tyr402 allotypes are in blue (013HH, ●; 022LM, ▲), and the His402 allotypes are in red (015LT, ○; 032KO, Δ).

In order to test the impact of zinc on the regulatory role of the Tyr402 and His402 FH allotypes, we performed assays of fluid-phase ammonium-inactivated C3 (C3u) cleavage by factor I and two allotypes of FH by zinc titration.^51^ Previous fluid-phase activity assays had been performed at significantly lower FH concentrations that included 45 μg/ml,^51^ 3 μg/ml,^52^ 30 μg/ml,^53^ and 0.3 μg/ml^54^ than the 0.235–0.81 mg/ml (1.5–5.3 μM) observed *in vivo*. Our assays with the FH allotypes were performed at 0.3 mg/ml (1.9 μM), which is comparable with both its physiological range and the heterozygous FH concentrations used previously.^21^ Thus, the α-chain of C3u was cleaved by factor I in the presence of the FH Tyr402 allotype without zinc to produce two major degradation fragments at apparent sizes of 45 kDa and 75 kDa (Fig. 9a), and the cleavage rate was significantly reduced in the presence of 200 μM zinc (Fig. 9b). The influence of zinc on this reaction for both FH allotypes was investigated at concentrations of 0 μM, 120 μM, and 200 μM (Fig. 9b). This revealed that the C3u cleavage rate was decreased by zinc, in good agreement with the decrease seen previously for heterozygous FH.^21^ No difference between the Tyr402 and the His402 allotypes was seen (Fig. 9c). If similar levels of FH oligomerisation with both allotypes accounted for the decrease in FH activity by blocking FH access to C3u and/or factor I,^21^ this would account for the observed reduced rate of C3u cleavage.

### Bioinformatics prediction of zinc binding sites on full-length FH

In order to validate the experimental data analyses above, we applied two independent bioinformatics strategies in order to predict potential tetrahedral zinc binding sites in FH. The first strategy utilised a distance-dependent, knowledge-based method that was based on known zinc binding sites. A total of 3705 proteins with Zn binding sites was identified in the Protein Data Bank (PDB), and these were screened to identify the residues that were within 0.3 nm of the metal site. Zinc binding sites at the interface between two protein surfaces generally contain Asp, His, and Glu side chains as zinc coordination sites.^46^ Cys residues were not included in this survey because there are no exposed Cys residues in full-length FH. Accordingly, a total of 830 zinc binding sites composed of His, Glu, and/or Asp residues only was identified and classified into 33 subgroups based on their compositions. A contact matrix (CM) was defined for each subgroup that is based on the mean separation ± 1 SD between the α-carbon atoms and that between the β-carbon atoms (Materials and Methods). This matrix was used to screen the FH domains in order to predict potential interface zinc binding sites. For this, crystal and NMR structures for 11 SCR domains and 9 homology-modelled structures that were available at the time of this work were used (Table 1a).^28,55–58^ A total of 202 possible partial zinc binding sites was identified as residue pairs or triplets that could act as one-half of a tetrahedral zinc interface binding site. In some cases, a pair or triplet of residues could form a partial zinc binding site with more than one coordination geometry (Table 1b). In order of probability, 31% of these sites were identified in SCR-6, which had 62.5 sites, followed by 12% in SCR-3, 10% in SCR-16, 7% sites in SCR-2, and 7% sites in SCR-14. Two or fewer predicted zinc binding sites were found in five SCR domains (Table 1a). All the triplets were located in SCR-6 or between SCR-6 and SCR-7 (Table 1b). The predicted highest number of zinc binding sites in SCR-6 agrees with the similar experimentally observed effect of zinc on the aggregation of both full-length FH and SCR-6/8. The predicted involvement of SCR-2, SCR-3, and SCR-16 would explain the ultracentrifugation experiments with SCR-1/5 and SCR-16/20 that suggest that other weak zinc binding sites may be present, assuming that these additional sites supplement the zinc-binding role of the His-tag. Even though the disease-related His402 residue may be a potential zinc-binding residue, His402 occurred in only 2 out of the 202 zinc binding sites predicted in FH. The Tyr402His polymorphism thus has a low probability of altering the interaction with zinc, and this agrees with the experimentally observed lack of difference between the Tyr402 and the His402 allotypes of FH and SCR-6/8 after zinc addition by ultracentrifugation and scattering.

The second bioinformatics strategy was based on submitting the 20 crystal, NMR, and homology SCR structures to web servers that predict metal binding sites in proteins (Materials and Methods). METSITE was developed using relative residue positions and does not require side-chain atoms to be present; therefore, this is applicable to low-resolution structures.^59^ It has a high sensitivity of 94.6% and a medium specificity of 47.8% for zinc. This method is based on recognizing residue environments in which a metal ion is likely to be found within 0.7 nm. These environments are represented by structural features such as residue β-carbon distances and solvent accessibility. When a 0.4-nm threshold was applied to the METSITE results, METSITE predicted that 26 out of 175 His, Asp, and Glu residues in FH were candidates for 13 predicted interaction sites with zinc. SCR-6 with five residues gave the highest total among the 20 SCR domains, followed by SCR-15 and SCR-20 with three each. A ranking based on the number of residues per zinc binding site (between 1.3 and 1.4) showed that SCR-6, SCR-7, SCR-8, SCR-15, and SCR-20 were the most likely domains to contain zinc binding sites.

**Fig. 10:**
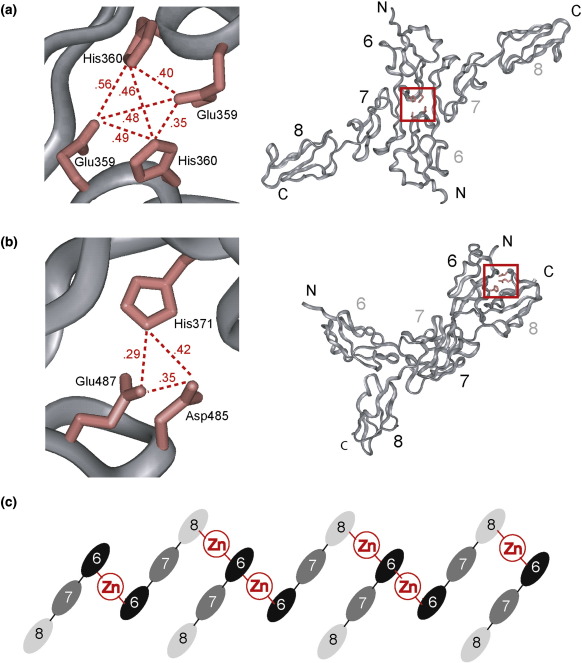
Predicted structural models for zinc binding to SCR-6/8. The two most likely predicted SCR-6/8 zinc sites from docking simulations are shown in (a) and (b), and a cartoon of the resulting putative zinc-induced SCR-6/8 aggregation is shown in (c). (a) A predicted zinc binding site is composed of two Glu359 and His360 side chains (Table 2) at the surface interface between two SCR-6 domains in the docked dimer. The left panel shows a close-up view of the zinc-binding residues, while the right panel shows the overall structure of the putative dimer. The distances between each zinc ligand atom (nitrogen or oxygen) are displayed in nanometers. (b) A second predicted zinc binding site is composed of a His371 side chain in SCR-6 of the first SCR-6/8 monomer and the Asp485 and Glu487 side chains in SCR-8 of the second SCR-6/8 monomer (Table 2). Other details follow (a). (c) A mechanistic model for the zinc-induced aggregation of SCR-6/8 based on the two possible zinc binding sites shown in (a) and (b) shows how the two zinc sites may form a daisy chain of SCR-6/8 that leads to aggregation and precipitation.

Given this outcome from both bioinformatics strategies, the crystal structure of SCR-6/8 (PDB code 2UWN) was examined to see whether tetrahedral zinc binding sites were possible in SCR-6/8 dimers. A total of 195 homodimeric structures for SCR-6/8 was created using four different protein docking servers (Materials and Methods). All 195 dimeric structures were subjected to zinc binding site prediction using the web servers METSITE and CHED (Materials and Methods). METSITE identified possible tetrahedral zinc binding sites in 193 out of 195 structures, while CHED with no activated filters identified possible zinc binding sites in 91 out of 195 structures. The difference is attributed to the higher sensitivity but lower selectivity of METSITE compared to CHED.^59,60^ The 10 most frequent possible zinc binding site residues in the 195 dimeric structures were Glu359, His360, His371, Asp370, Asp485, His332, His417, Glu462, Glu487, and Asp497 in order of frequency (Table 2). Interestingly, His402 was not included in those 10 residues. This provides an explanation with the experimental observations of the lack of difference in oligomerisation between the two SCR-6/8 allotypes in the presence of zinc. In order to rationalise the outcome of these searches, we analysed the 195 putative dimeric structures with RMSDCLUST (Materials and Methods), which superposes every pair of structures and identifies clusters of similar structures. Nine different zinc binding sites were found in seven clusters that each contained 18, 15, 4, 2, 3, 4, and 5 SCR-6/8 dimer structures, together with a single outlier that did not belong to any cluster. Only two out of the nine possible zinc binding sites possessed all the residues identified as the 10 most frequent possible zinc-binding residues. One of these binding sites involved Glu359 and His360 on each of two SCR-6/8 monomers (Fig. 10a), and the other involved His371 on one SCR-6/8 monomer and Asp485 and Glu487 on the other monomer (Fig. 10b). If zinc cross-links pairs of SCR domains in tetrahedral coordinations, the predictions in Fig. 10a and b lead to a schematic cross-linking cartoon in which adjacent pairs of two SCR-6 domains or SCR-6 and SCR-8 domains form contacts with each other. The indefinite self-association suggested in Fig. 10c shows how zinc may cross-link SCR-6/8 molecules to form large aggregates. As a control of this predictive approach, the same procedure was performed with three protein structures with known zinc binding sites (Table 2). These were human interferon-β, the complex of human growth hormone and prolactin, and an archaeal cytochrome P450. All the known zinc-binding residues in the three known protein structures were correctly included in the top 10 most frequently occurring residues, and this outcome supports the credibility of this predictive method for FH SCR-6/8.

## Conclusions

This investigation of FH–zinc interactions followed the observation that sRPEds and the Bruch's membrane in postmortem human tissues contain unexpectedly high concentrations of zinc, including abundant bioavailable ions (ionic and/or loosely protein bound).^42^ Zinc accumulation was especially high in the maculae of eyes with AMD.^42^ There, the total extracellular zinc was measured to be hundreds of parts per million, which is equivalent to zinc concentrations of several millimolar. Most of the zinc will be inaccessible for reason of its tight binding to components within drusen. The evidence for bioavailability arises from the use of fluorescent probes because these only bind to bioavailable zinc.^42^ The bioavailability of only 1% of this millimolar zinc in the extracellular space is sufficient to cause our FH–zinc complexes to aggregate.^21^ While further experimental evidence for this level of zinc bioavailability *in vivo* in relation to AMD will be required, metal-induced protein aggregation has been shown to be significant in other degenerative diseases such as in amyloid and prion diseases.^61^ Under pathophysiological conditions, normal physiological/chemical buffering mechanisms will be compromised, and the basal level of nanomolar or picomolar free zinc ion concentrations can increase substantially.^62^ Our zinc concentrations from 10 μM upwards, at which we observed the effects with FH, therefore have biomedical significance.

Such interactions of extracellular proteins with micromolar concentrations of zinc ions (as illustrated here for FH–zinc) are deemed physiologically relevant based on total zinc concentrations. During neuronal activity, Zn is released into the synaptic cleft and can reach concentrations of 300 μM.^63^ Zinc inhibition constants in micromolar ranges for kallikreins and carboxypeptidase A are also likely to be physiologically relevant because these enzymes are secreted from tissues that also secrete zinc ions. It is however stressed that this is not the case for intracellular proteins. The total cellular zinc concentration can be as high as several hundred micromolars, especially in the nervous system (which includes the eye).^64^ In these cells, the cytosolic bioavailable free zinc ion concentration is in picomolar ranges, and zinc-binding proteins have very low *K*_d_ values to match these.^65,66^

Overall, this study has located a major FH zinc binding site to lie within the functionally important SCR-6/8 domains, and this accounts for the inhibition of FH activity by zinc. Our two earlier studies showed that heterozygous FH aggregated in the presence of five transition metals at physiological FH concentrations, particularly at zinc and copper concentrations of ≥ 10 μM.^20,21^ Zinc has the strongest effect on FH and inhibits its function. In this study, using ultracentrifugation and X-ray scattering, we have shown that similar zinc-induced aggregation occurs for both the homozygous forms of FH with either the Tyr402 or the disease-related His402 allotype. The FH Val62 or Ile62 polymorphism (which is also disease related) also has no influence on zinc binding effects. Therefore, zinc-induced aggregation is independent of FH genetic heterogeneity. Based on ultracentrifugation and scattering data, which were validated by bioinformatics predictions, we showed that SCR-6/8 is a major locus for zinc-induced aggregation through the presence of partial zinc binding sites at the FH surface. These sites can be cross-linked in the presence of zinc to form large oligomers. This daisy-chained cross-linking scheme is explained in terms of putative SCR-6/SCR-6 interactions that alternate with putative SCR-6/SCR-8 interactions (Fig. 10c). Control experiments with the other two functionally active regions of FH at SCR-1/5 and SCR-16/20 showed that both regions may also aggregate in the presence of zinc. However, further controls with added nickel indicated that this is less significant than that at SCR-6/8. This study now explains the zinc-induced formation of FH oligomers for the first time in terms of multiple weak surface zinc binding sites that are located primarily within the SCR-6/8 region. The compact nature of these FH oligomers (Fig. 8) is explained by zinc binding at a central location in FH within SCR-6/8. The proximity of SCR-6/8 to the C3b-binding region of FH at SCR-1/5 explains why zinc binding inhibits FH; in addition, the SCR-6/8 domains bind to heparin and C-reactive protein. It was interesting that the Tyr402 or His402 allotype has no significant influence on zinc-induced aggregation, given that His402 might have been a potential surface zinc-binding residue.

FH is a major complement regulator in blood and is expressed and secreted by many different cell types including the RPE.^67^ The major physiological ligands of FH include C3b and its C3d fragment, heparin and other glycosaminoglycans, and C-reactive protein and FH self-association, in addition to zinc binding.^30,31^ All these ligands bind weakly to FH with micromolar affinities, and this is consistent with the relative micromolar abundance of FH in serum. The FH–zinc interaction has already been discussed previously.^21^ Since that 2008 study, more recent studies show that C3b and C3d bind with *K*_d_ values of 3.5–14 μM and 2.6 μM, respectively,^12,68^ C-reactive protein binds with a *K*_d_ value of 4–15 μM,^13^ and FH dimerises with a *K*_d_ value of 28 μM.^19^ If FH is found at 0.6 mg/ml in serum, this means that 11% of FH exists as dimers. If C3b occurs at 1 mg/ml, 29% of FH will be bound to C3b if the *K*_d_ value is 3.5 μM. If C-reactive protein occurs at the acute-phase level of 0.4 mg/ml, 32% of FH will be bound to C-reactive protein if the *K*_d_ value is 4.2 μM. These calculations assume that no other factors in serum influence these equilibria, which have been considered separately from each other for the purpose of illustration here. In comparison to these physiological interactions, the pathophysiological interaction between FH and zinc is characterised by comparable *K*_d_ values at approximately 10 μM, implying that FH will be 50% bound to zinc if bioavailable zinc is present at 10 μM. However, most of the zinc in plasma is bound to proteins such as human serum albumin (HSA),^69^ and only a small proportion of this zinc is bioavailable in plasma.^65^ Potentially higher bioavailable zinc levels may exist in Bruch's membrane (see below). Because two of the most frequently predicted zinc-binding residues, His360 and His371 (Table 2), have been implicated in glycosaminoglycan binding to FH,^57^ it is possible that zinc binding to FH can perturb the binding of glycosaminoglycans to FH.

The comparison of zinc binding to FH and HSA is of interest. HSA is a three-domain protein that circulates in serum at reference levels between 30 and 50 mg/ml (450–750 μM).^69^ HSA binds 84% of plasma zinc, and α2-macroglobulin binds 15%.^66^ In HSA, a zinc binding site was identified at the protein surface interface between domain I at His67 and Asn99 and domain II at His247 and Asp249 (PDB code 1AO6) that is conserved in almost all mammalian species.^69,70^ Here, asparagine is a rare zinc-binding ligand. Being stereochemically well-defined, the *K*_d_ for zinc binding to HSA is lower at 1 μM compared to that of approximately 10 μM for zinc binding to FH. The larger *K*_d_ value for the FH–zinc interaction is explained by the lack of proximity in monomeric FH of appropriate pairs of the SCR domains that possess potential zinc-binding residues. Given the HSA  concentration of 450–750 μM and the serum zinc concentration of 12.5 μM, where HSA binds 84% of plasma zinc, the presence of HSA will reduce the level of bioavailable zinc, and accordingly, there is little risk of FH aggregation with zinc in plasma. The total zinc level in plasma remains steady at 14.7 μM even after a daily diet supplement with 80 mg zinc in the AREDS trials.^43,71^ Therefore, since bioavailable zinc in plasma occurs in a 20- to  210-pM range,^65^ normal zinc levels in plasma are too low to induce FH oligomerisation.

Human ocular tissues contain unusually high concentrations of zinc.^40^ Zinc appears to be essential for the normal function of the retina, but its exact role is not clear.^41^ In the RPE–choroidal complex, zinc is expected to be mostly bound to melanin, metallothionein, and other zinc-binding proteins, and it has been proposed that less than 10% of zinc in the retina is free or chelatable.^40,41,72^ RPE cells can accumulate zinc following oral supplementation and retain it longer than any other tissues in the body, but zinc might be released from the RPE through cellular damage.^72^ The released zinc could then be available in the Bruch's membrane or be entrapped in sRPEds and contribute to the several hundred parts per million of zinc (mM) observed in sub-RPE deposits.^41,42^ This RPE-released zinc might be sufficient to induce a localised aggregation of FH that has been recruited to the location because of inflammation associated with RPE damage. The comparison above with the *K*_d_ values for FH interactions with its other ligands shows that the regulatory balance of FH activity in terms of interactions with C3b, heparin, and C-reactive protein can be disturbed by zinc binding. The main interaction of zinc with the SCR-6/8 regions, which is a locus for interactions with heparin and C-reactive protein, indicates that FH function with these two ligands will be perturbed if sufficient excess zinc is available. In addition, the indefinite self-association of FH at SCR-6/8 is expected to block the binding of the neighbouring FH SCR-1/4 domains to C3b. Such a mechanism can contribute to both the formation of sRPEds and the inhibition of the regulatory function of FH to cause inflammation and host cell damage.

Daily supplements with 80 mg zinc, 2 mg copper, vitamins C and E, and β carotene are recommended by AREDS as a means of reducing the risk for progression to advanced AMD.^43,44^ Interestingly, the AREDS-recommended zinc supplements with antioxidants were recently correlated with a greater reduction in progression to advanced AMD in patients who are homozygous for the FH Tyr402 allotype (wild type; 34% of cases) compared to patients who are homozygous for the FH His402 allotype (AMD risk; 11% of cases).^45^ Our current study offers no direct explanation for this observation because no significant difference in zinc-induced FH aggregation was seen between the Tyr402 and the His402 allotypes of FH and SCR-6/8 (Figs. 2 and 7).

The most common coordination geometry of zinc binding sites is tetrahedral.^46^ Here, our bioinformatics analyses showed that 3705 zinc binding coordinations were available for analysis from the PDB. Protein interface zinc binding sites are primarily supplied by His, Glu, and Asp residues, but sites containing Cys are also found (not relevant to FH because it contains no free Cys residues), and β-sheet secondary structures predominately contribute to zinc binding sites. Zinc binding is able to induce protein self-association or link two different proteins.^46^ Our previous discussion of potential FH zinc binding sites^21^ is now clarified by the abundance of partial zinc sites that were predicted using a distance-based algorithm, suggesting that multiple weak zinc binding sites exist in FH if zinc cross-linked FH. This outcome predicts that a range of different zinc-induced oligomers will form at several sites. This multiplicity of oligomers is in good agreement with the observation of many overlapping peaks in the ultracentrifugation *c*(*s*) size distribution analyses (i.e., the opposite of the formation of fewer well-resolved signals such as the nine peaks observed for FH alone^8,19^) and also with the formation of indefinite oligomers at high zinc concentrations. The prediction of multiple sites also explains better why different types of FH oligomers were observed with different metals such as copper.^21^ Finally, the predictions were also informative in that they explained the lack of difference seen between the SCR-6/8 Tyr402 and the His402 allotypes because His402 was not predicted to be a significant zinc-binding ligand. These analyses provide insight into the weak binding of zinc to other plasma proteins. It is possible that the outcome of this FH–zinc study will be applicable to other plasma proteins. For example, we have observed that zinc will induce oligomer formation in C3 (K. Li and S.J.P., unpublished data).

In summary, our results contribute to a better understanding of how zinc might be involved in two distinct and different ways that contribute to AMD.^73^ In early AMD, zinc release may trigger sRPEd formation and contribute to the growth of these deposits through oligomerisation of proteins including FH. This will lead not only to deposit formation but also to uncontrolled inflammation, the two hallmarks for the development of this blindness condition. In late AMD, however, zinc supplementation could slow disease progression through a currently unidentified mechanism. This, however, is unlikely to involve FH oligomerisation.

## Materials and Methods

### Protein purification of FH and FH SCR-6/8

Human blood was obtained from 48 anonymous, genotyped, healthy volunteers following ethical approval. Direct DNA sequencing of the PCR product of the FH gene was used to identify the Tyr402His and Val62Ile FH polymorphisms in each volunteer. Four homozygous FH Tyr402 allotypes (coded 026II, 022LM, 018DK, and 016CC) and four FH His402 allotypes (coded 027BA, 032KO, 030TJ, and 033NV) were purified using monoclonal affinity chromatography with an MRC-OX23 Sepharose column.^19,74^ The four Tyr402 allotypes were homozygous for Ile62, homozygous for Val62, heterozygous for Val62/Ile62, and homozygous for Val62 in that order, while all four His402 allotypes were homozygous for Val62. Native factor I was purified from a pool of just-outdated anonymised human plasma using another monoclonal affinity MRC-OX21 Sepharose column for factor I.^19,74^ Bound homozygous FH and native factor I were each eluted from the columns using 3 M MgCl_2_, pH 6.9, and then each was dialysed in Hepes buffer (10 mM Hepes and 137 mM NaCl, pH 7.4) in the presence of 0.5 mM EDTA to remove Mg^2+^. The MRC-OX23 column was washed with guanidine (0.2 M Tris and 4 M guanidine–HCl, pH 8.0) after each FH preparation to avoid allotype cross-contamination. IgG contaminants were removed by passing the FH and factor I purifications through a Hitrap™ Protein G HP. Nonspecific aggregates and HSA were removed by gel filtration on a Superose™ 6 prep grade XK 16/60 size-exclusive column. Native C3 was purified from fresh plasma by ion-exchange chromatography, and haemolytically inactive C3 in which the reactive thioester is hydrolysed (C3u) was prepared by incubating native C3 in 200 mM hydrazine at 37 °C for 1 h.^75^ The recombinant FH fragment SCR-6/8 Tyr402 was expressed in BL21 (DE3) *Escherichia coli* cells using a pET21ab vector system, while SCR-6/8 His402 was expressed in BL21 (DE3)pLysS *E. coli* cells using a pET14b vector system. SCR-6/8 was extracted from inclusion bodies, solubilised, and refolded. The refolded SCR-6/8 was purified by using ion-exchange chromatography with a Hitrap™  5-ml Heparin HP column (A.M. and S.J.P., unpublished data).^17^ The purifications of SCR-1/5 and SCR-16/20 are described elsewhere.^18^ Proteins were dialysed in Hepes buffer for zinc experiments because Hepes does not interact with zinc. Protein concentrations were determined from absorption coefficients of 16.2 for full-length FH Tyr402 and 16.1 for full-length FH His402, 12 for factor I, 9.4 for C3u, 17.2 for SCR-1/5, 22.7 for SCR-6/8 Tyr402, 22.1 for SCR-6/8 His402, and 15.7 for SCR-16/20 (1%, 280 nm,  1-cm path length).^6,7,18,76,77^ The integrity of protein samples was routinely checked by SDS-PAGE before and after scattering and ultracentrifugation experiments.

### Sedimentation velocity data collection and analyses

AUC data were obtained on two Beckman XL-I instruments equipped with AnTi50 or AnTi60 rotor using two-sector cells with column heights of 12 mm at rotor speeds of 50,000 rpm and 60,000 rpm. Sedimentation velocity experiments at 20 °C were performed for the two FH Tyr402 allotypes (026II and 022LM) and the two His402 allotypes (027BA and 032KO) at 0.7–0.8 mg/ml, which were titrated with ZnSO_4_ at concentrations of 0.2 μM, 2 μM, 6 μM, 20 μM, 60 μM, 120 μM, and 200 μM. SCR-1/5 at 0.3 mg/ml was titrated with ZnSO_4_ at concentrations of 0 μM, 2 μM, 6 μM, 20 μM, 60 μM, 200 μM, and 600 μM. The Tyr402 and His402 allotypes of SCR-6/8 at 0.3 mg/ml were titrated with ZnSO_4_ at concentrations of 0.2 μM, 2 μM, 6 μM, 20 μM, 60 μM, 200 μM, 300 μM, and 600 μM. SCR-16/20 at 0.1 mg/ml was titrated with ZnSO_4_ at concentrations of 0 μM, 2 μM, 20 μM, 200 μM, and 600 μM. Data analyses were performed using the SEDFIT software (version 11.71).^78,79^ The distribution analyses *c*(*s*) provided size and shape data by directly fitting the boundary Lamm equation to 350 scans for FH, 480 scans for SCR-1/5, 640 scans for SCR-6/8, and 440 scans for SCR-16/20. The *c*(*s*) analyses were based on a fixed resolution of 200 and floated the meniscus, the bottom of the cell, the baseline, and the average frictional ratio *f*/*f*_o_ starting from 1.781 for FH and from 1.200 for SCR-6/8 until the overall root-mean-square deviation (RMSD) and fits between the observed and the calculated sedimentation boundaries were satisfactory. The *f*/*f*_o_ values were fixed at 1.45 for SCR-1/5, 1.35 for the SCR-16/20 monomer, and 1.58 for the SCR-16/20 dimer.^18^ Monomer and oligomers were quantitated using the integration function in the *c*(*s*) analyses. The percentage fraction of monomer and oligomers of full-length FH was derived by assuming that the sum of the signal intensities of monomer and oligomer was 100%. Other details are described elsewhere.^18,19^

### X-ray scattering data collection and analysis

X-ray scattering data were acquired for the FH allotypes in one beam session and for the SCR-6/8 allotypes in a second beam session on Beamline ID02 at the European Synchrotron Radiation Facility (Grenoble, France) operating with a ring energy of 6.0 GeV in 4-bunch mode and 16-bunch mode in the two beam sessions, respectively, to reduce the incident flux.^80^ Storage ring currents were 29–43 mA in the first beam session and 56–91 mA in the second beam session. The sample–detector distances were 3 m for the first beam session and 1.5 m for the second beam session. Potential radiation damage was eliminated by the continuous movement of the sample in a flow cell during beam exposure, the use of 10 time frames of duration between 0.1 s  and 0.25 s  each during each acquisition, and on-line checks for the absence of radiation damage at low *Q*. In the first beam session, the FH Tyr402 allotypes 018DK and 016CC and the His402 allotypes 030TJ and 033NV were studied at 0.9 mg/ml (5.8 μM) with ZnSO_4_ at concentrations of 0.2 μM, 2 μM, 6 μM, 20 μM, 60 μM, 120 μM, 200 μM, and 600 μM. In the second beam session, the Tyr402 and His402 allotypes of SCR-6/8 were studied at 0.2 mg/ml (9.7 μM) with ZnSO_4_ at concentrations of 0.2 μM, 2 μM, 6 μM, 20 μM, 60 μM, 120 μM, 200 μM, 300 μM, and 600 μM. All the measurements were performed in Hepes buffer (10 mM Hepes and 137 mM NaCl, pH 7.4). Other details including data reduction are described elsewhere.^17,18^

In a given solute–solvent contrast, the radius of gyration *R*_G_ corresponds to the mean square distance of scattering elements from their centre of gravity and is a measure of structural elongation. Guinier analyses at low *Q* give the *R*_G_ value and the forward scattering at zero angle *I*(0) from the expression:^50^

$$\text{ln}\;I\left(Q\right)\text{=ln }I\left(\text{0}\right)-R_{G}^{2}Q^{\text{2}}\text{/3}$$

This expression is valid in a *QR*_G_ range of up to 1.5. The *I*(0)/*c* value (*c* is the protein concentration in milligrams per milliliter) is proportional to the relative molecular mass *M*_r_. If the structure is elongated, the mean cross-sectional radius of gyration *R*_XS_ and the cross-sectional intensity at zero angle [*I*(*Q*)*Q*]_*Q*__ → 0_ are determined from Guinier analyses in a *Q* range larger than that used for the *R*_G_ determination:^50^

$$\text{ln}\left[I\left(Q\right)Q\right]\text{=ln}{\left[I\left(Q\right)Q\right]}_{Q\to \text{0}}-R_{\text{XS}}^{\text{2}}Q^{\text{2}}\text{/2}$$

The Guinier analyses were performed using the interactive PERL script program SCTPL7 (J. T. Eaton and S.J.P., unpublished software) on Silicon Graphics OCTANE Workstations.

Indirect transformation of the *I*(*Q*) curve measured in reciprocal space into real space gives the distance distribution function *P*(*r*) and was carried out using the program GNOM:^81^

$$P\left(r\right)=\frac{1}{2\pi^{2}}\int_{o}^{\infty }I\left(Q\right)Qr\;\sin\left(Qr\right)\text{d}Q$$

*P*(*r*) corresponds to the distribution of distances *r* between volume elements. This offers an alternative calculation of the *R*_G_ and *I*(0) values that is based on the full scattering curve *I*(*Q*), and not that at low *Q*. It also gives the most frequently occurring distance *M* and the maximum dimension of the macromolecule *L*. For FH at 0.9 mg/ml titrated with zinc, the X-ray curves utilised up to 344 data points for *Q* between 0.08 nm^− 1^ and 1.60 nm^− 1^. Other details are described elsewhere.^6,17–19^

### Fluid-phase activity assays

To determine activities, we incubated the reaction mixtures containing 0.3 mg/ml FH, 0.3 mg/ml C3u, and 0.003 mg/ml factor I in a water bath at 37 °C in three concentrations of ZnSO_4_ (0 μM, 120 μM, and 200 μM). The functions of two pairs of the two allotypes of FH were tested. The FH Tyr402 allotype 013HH and the FH His402 allotype 015LT were tested with 0 μM and 200 μM zinc, and the FH Tyr402 allotype 022LM and the His402 allotype 032KO were tested with 120 μM zinc. At timed intervals,  5-μl aliquots were removed for reducing SDS-PAGE analyses. Three blank controls were used, namely, 0.3 mg/ml C3u; 0.3 mg/ml C3u, and 0.3 mg/ml FH and 0.3 mg/ml C3u, 0.3 mg/ml FH, and zinc. The C3 α-chain band densities from SDS-PAGE were measured using the gel analysis system SYNGENE (Synoptics Ltd., Cambridge, UK). The C3 α-chain cleavage was referenced to the averaged density of the uncleaved bands in the controls.

### Molecular prediction of surface zinc binding sites

Two independent database analyses were used to predict partial protein-interface-type zinc binding sites on protein surfaces. In the first one, a data set of 3705 Zn-binding protein crystal structures with resolutions better than 0.3 nm was identified from the PDB. Residues that were located within 0.3-nm distance of the zinc atom were assigned as putative zinc-coordinating residues using a PERL script to scan the PDB. Of the 4883 putative zinc binding sites that were identified, a total of 830 zinc binding sites composed of His, Glu, and/or Asp as zinc coordination residues was analysed and classified into 33 subgroups based on the amino acid composition of each site. In each subgroup, the distance *x* between the C^α^ atoms *i* and that between the C^β^ atoms *j* of each zinc-coordinating residue were calculated as a residue pair type *x*(*i*,*j*) and stored in a CM (contact matrix). For each residue pair in CM, the average distance x̄ and its standard deviation σ were calculated. An algorithm was implemented to screen each protein structure of interest for residues that form putative partial Zn binding sites. For a given protein structure, solvent-exposed Asp, His, and Glu side chains were identified. A residue is considered exposed if its side chain has a relative surface accessibility greater than 20%. The geometric distances between pairs and triplets of C^α^ atoms and C^β^ atoms of the putative zinc-coordinating residues were calculated. If these distances fall within the distance cutoff x̄ ± σ, then the pair or triplet of residues become considered as possible zinc-coordinating residues. Using this algorithm, we screened 11 experimental SCR crystal or NMR structures for SCR-1/2, SCR-2/3, SCR-5, SCR-6/8, SCR-15/16, and SCR-19/20^28,55–57^ and 9 homology-modelled SCR structures for SCR-4, SCR-9, SCR-10, SCR-11, SCR-12, SCR-13, SCR-14, SCR-17, and SCR-18.^28^

The second prediction of partial metal binding sites in FH was carried out using web servers. In this, the 20 experimental and homology SCR structures in FH were evaluated using METSITE†,^59^ which uses a set of neural network classifiers trained to identify potential cation sites. Following this analysis, potential zinc sites at dimer interfaces were studied. To form FH dimer structures, we docked the SCR-6/8 His402 crystal structure (PDB code 2UWN) using four different docking approaches available as web servers including ClusPro‡, HEX§, PatchDock||, and RosettaDock¶.^82–87^ These four docking programs were selected according to their performance in the Critical Assessment of Predicted Interactions surveys.^88^ These possible dimeric structures were tested again by METSITE and another web server, CHED,^60^ to detect possible tetrahedral zinc binding sites at the protein interface in the dimeric structures. The CHED web server^a^ searches for Cys, His, Glu, and Asp residues with atoms within several specified distances to each other and a 0.27-nm distance to the putative zinc atom position. If the distances do not match, an independent rotamer library is used to find an appropriate fit configuration. The sites found in CHED are filtered by a decision tree and a support vector machine to verify their suitability. The dimeric structures that possess zinc binding sites predicted by METSITE and CHED were clustered using the program RMSDCLUST (D.T.J., unpublished software) to group similar structures. RMSDCLUST carries out rigid-body superposition between each pair of dimeric structures, computes the α-carbon RMSD, and then clusters all pairs of structures within an adjustable RMSD threshold (0.6 nm in this case). To validate the methods, we performed the same predictions with three zinc binding structures as benchmarks, namely, human interferon-β (PDB code 1AU1), the complex of human growth hormone and prolactin (PDB code 1BP3), and thermophilic cytochrome P450 from *Sulfolobus solfataricus* (PDB code 1F4T).^89–91^

## Figures and Tables

**Table 1:** Prediction of partial zinc binding sites in FH

a. Distribution of predicted zinc coordination sites in 20 SCR domains		
Domain	Structure	Sites^a^
SCR-1	2RLP	6
SCR-2	2RLP, 2RLQ , 2RLQ	14.5
SCR-3	2RLQ	23.5
SCR-4	Homology	2
SCR-5	NMR structure	0
SCR-6	2UWN	62.5
SCR-7	2UWN	8.5
SCR-8	2UWN	1
SCR-9	Homology	5
SCR-10	Homology	4
SCR-11	Homology	14
SCR-12	Homology	9
SCR-13	Homology	8
SCR-14	Homology	4
SCR-15	1HFH	7.5
SCR-16	1HFH	19.5
SCR-17	Homology	0
SCR-18	Homology	1
SCR-19	2G7I	7.5
SCR-20	2G7I	4.5


b. Possible predicted zinc binding sites in the FH fragment SCR-6/8^b^		
Residues	Location	Possible zinc coordination
D326–D329	SCR-6	DDH, DDHH
D329–H332	SCR-6	DDHH, DHHH
H332–D358	SCR-6	DDDH, DDHH, DEHH, DH, DHH, DHHH
H332–D358–E359	SCR-6	DEHH
H332–D358–H360	SCR-6	DDHH, DEHH, DHH, DHHH
H332–D358–E362	SCR-6	DDHH, DEHH
H332–E359	SCR-6	EH, EHH, EHHH
H332–H360	SCR-6	DDHH, DEHH, DHH, DHHH, EHH, HH, HHH
H332–H360–E362	SCR-6	DEHH
H332–H360–E433	SCR-6/7	DEHH
H332–E362	SCR-6	DEHH, EHH, EHHH
H332–E433	SCR-6/7	EHHH, DEH, DEHH
H337–E338	SCR-6	EH, EHHH
D358–E359	SCR-6	DE
D358–H360	SCR-6	DDHH, DH, DHH, DHHH
D358–H360–E362	SCR-6	DEHH
H360–E362	SCR-6	DEHH, EEEH, EEHH, EH, EHHH
H360–E433	SCR-6/7	EHH, DEHH, EHHH
D370–H371	SCR-6	DDHH, DEHH, DHH
D370–H371–H373	SCR-6	DDHH, DHH, DHHH
D370–H373	SCR-6	DDHH, DHH, DHHH
H371–H373	SCR-6	DHHH, EEHH, EHH, HHH, HHHH
H373–D377	SCR-6	DHHH, DDHH
E395–H402	SCR-7	EHH, EHHH
E395–H417	SCR-7	DEHH, EHH, EHHH
D454–E456	SCR-8	DDH, DDE

a Zinc coordination sites that are shared between two adjacent SCR domains are counted as 0.5 per domain.

b His402 occurred in 2 of the 76 possible zinc coordinations in SCR-6/8. Zinc coordinations that contain two or three residues also contain two or one H_2_O molecules, respectively, to form the required tetrahedral geometry with zinc.

**Table 2:** The most likely zinc interaction ligands in FH SCR-6/8 and three protein benchmarks

	FH SCR-6/8 (PDB code 2UWN)	Human interferon β (PDB code 1AU1)	Complex of human growth hormone and prolactin (PDB code 1BP3)	Archaeal cytochrome P450 (PDB code 1F4T)
Top 3	Glu359	Glu42	His18^a^	Asp52
	His360	His93^a^	Glu174^a^	His340
	His371	His121^a^	Asp387^a^	Glu342
Top 5	Asp370	Glu85	His21	Asp42
	Asp485	His131	His388^a^	His178^a^
Top 10	His332	Glu81	Glu56	Glu48
	His417	His97^a^	Asp171	Asp52
	Glu462	Glu103	Glu245	Glu139^a^
	Glu487		Glu292	Glu274
	Asp497		Glu345	Asp294

a The original ligands in the three benchmarked structures 1AU1, 1BP3, and 1F4T.

## Footnotes

† http://bioinf.cs.ucl.ac.uk/metsite

‡ http://cluspro.bu.edu/login.php

§ http://www.csd.abdn.ac.uk/hex_server/

|| http://bioinfo3d.cs.tau.ac.il/PatchDock/

¶ http://rosettadock.graylab.jhu.edu/

a http://ligin.weizmann.ac.il/~lpgerzon/mbs4/mbs.cgi
